# DQB1*0602 rather than DRB1*1501 confers susceptibility to multiple sclerosis-like disease induced by proteolipid protein (PLP)

**DOI:** 10.1186/1742-2094-9-29

**Published:** 2012-02-08

**Authors:** Nathali Kaushansky, Daniel M Altmann, Chella S David, Hans Lassmann, Avraham Ben-Nun

**Affiliations:** 1 Department of Immunology, The Weizmann Institute of Science, Rehovot 76100, Israel; 2 Human Disease Immunogenetics Group, Section of Infectious Diseases and Immunity, Department of Medicine, Imperial College, Hammersmith Hospital, London, UK; 3 Department of Immunology, Mayo Clinic, Rochester, MN, USA; 4 Center for Brain Research, Department of Neuroimmunology, Medical University of Vienna, Vienna, Austria; 5 Department of Immunology, The Weizmann Institute of Science, P.O. Box 26 Rehovot 76000, Israel

**Keywords:** EAE/MS, Antigens/Peptides/Epitopes, Neuroimmunology, T Cells, MHC, HLA-Tg mice

## Abstract

**Background:**

Multiple sclerosis (MS) is associated with pathogenic autoimmunity primarily focused on major CNS-myelin target antigens including myelin basic protein (MBP), proteolipidprotein (PLP), myelin oligodendrocyte protein (MOG). MS is a complex trait whereby the HLA genes, particularly class-II genes of HLA-DR15 haplotype, dominate the genetic contribution to disease-risk. Due to strong linkage disequilibrium in HLA-II region, it has been hard to establish precisely whether the functionally relevant effect derives from the DRB1*1501, DQA1*0102-DQB1*0602, or DRB5*0101 loci of HLA-DR15 haplotype, their combinations, or their epistatic interactions. Nevertheless, most genetic studies have indicated DRB1*1501 as a primary risk factor in MS. Here, we used 'HLA-humanized' mice to discern the potential relative contribution of DRB1*1501 and DQB1*0602 alleles to susceptibility to "humanized" MS-like disease induced by PLP, one of the most prominent and encephalitogenic target-antigens implicated in human MS.

**Methods:**

The HLA-DRB1*1501- and HLA-DQB1*0602-Tg mice (MHC-II^-/-^), and control non-HLA-DR15-relevant-Tg mice were immunized with a set of overlapping PLP peptides or with recombinant soluble PLP for induction of "humanized" MS-like disease, as well as for ex-vivo analysis of immunogenic/immunodominant HLA-restricted T-cell epitopes and associated cytokine secretion profile.

**Results:**

PLP autoimmunity in both HLA-DR15-Tg mice was focused on 139-151 and 175-194 epitopes. Strikingly, however, the HLA-DRB1*1501-transgenics were refractory to disease induction by any of the overlapping PLP peptides, while HLA-DQB1*0602 transgenics were susceptible to disease induction by PLP139-151 and PLP175-194 peptides. Although both transgenics responded to both peptides, the PLP139-151- and PLP175-194-reactive T-cells were directed to Th1/Th17 phenotype in DQB1*0602-Tg mice and towards Th2 in DRB1*1501-Tg mice.

**Conclusions:**

While genome studies map a strong MS susceptibility effect to the region of DRB1*1501, our findings offer a rationale for potential involvement of pathogenic DQ6-associated autoimmunity in MS. Moreover, that DQB1*0602, but not DRB1*1501, determines disease-susceptibility to PLP in HLA-transgenics, suggests a potential differential, functional role for DQB1*0602 as a predisposing allele in MS. This, together with previously demonstrated disease-susceptibility to MBP and MOG in DRB1*1501-transgenics, also suggests a differential role for DRB1*1501 and DQB1*0602 depending on target antigen and imply a potential complex 'genotype/target antigen/phenotype' relationship in MS heterogeneity.

## Background

Multiple sclerosis (MS) is a disease of the human central nervous system (CNS), characterized by perivascular inflammation, accompanied by primary demyelination and axonal damage. It is believed to result from autoimmune mechanisms leading to destruction of myelin, presumably initiated by abnormally activated T cells that recognize CNS components in MS patients. The pathogenic autoimmunity in MS appears to be associated with complex immune reactivity directed against several CNS-specific and non CNS-specific components [1,2]. Many of the primary target antigens detected in T cell responses of MS patients share identity with those CNS antigens demonstrated to cause overt, clinical EAE in laboratory animals. Thus far, several myelin proteins, myelin basic protein (MBP), proteolipid protein (PLP), and more recently, myelin oligodendrocyte glycoprotein (MOG), myelin-oligodendrocytic basic protein (MOBP), oligodendrocye specific protein (OSP) [1,2], and the neuronal components [(β-synuclein (βSyn), neurofilament light (NF-L)] [3,4] fulfill these criteria. In attempts to establish a molecular etiology of MS that both explains the genetic associations and potentiates specific therapeutic interventions, defining the potentially pathogenic epitopes of major MS-related CNS target antigens, in the context of their HLA restricting genes/alleles, and characterization of the corresponding responder T cells will be essential.

Both genetic and environmental factors have been shown to contribute to the pathogenesis of MS. Despite extensive studies on the role of genetic and environmental factors that have been associated with the etiology of MS [2], the effects of other potential MS-risk factors are dwarfed by the contribution from the HLA class II region [5-8]. In recent genome-wide association studies, several HLA and non-HLA genes have been associated with the disease, with the HLA-class II genes, particularly those of the HLA-DR15 haplotype (HLA-DQB1*0602- HLA-DQA1*0102; HLA-DRB1*1501; HLA-DRB5*0101) bearing the strongest association to MS [9,10]. The HLA DR15 haplotype, which is most prevalent among Caucasian MS patients, encodes three functional class II heterodimers, DR15 (the DRA1*0101/DRB1*1501 pair), DRB5 (the DRA1*0101/DRB5*0101 pair), and DQ6 (DQA1*0102/DQB1*0602 pair). Due to extensive linkage disequilibrium across the HLA-II region [11], fine-genetic mapping studies could not unequivocally establish whether the functionally relevant effect on MS derives from DRB1*1501, DQA1*0102, DQB1*0602, or DRB5*0101 loci of HLA-DR15, their co-expression, or from their epistatic interactions [7,12]. Candidate gene association studies for HLA association in MS tend to indicate DRB1*1501 (with the DQB1*0602 allele in linkage disequilibrium) as the primary risk factor for increased susceptibility [13-15]. Relatively few studies, often in smaller defined ethnic groups, indicate an effect of DQB1*0602 independent of DRB1*1501 [16,17], or of DRB1*1501 independent of DQB1*0602 [18]. In functional, in vitro studies of T-cell clones and lines from MS patients, emphasis has been largely on DR15-restricted T cells specific for various myelin components, particularly MBP [19,20]. There have been rare studies of HLA-DQ6-restricted T cells clones in MS patients, including cells specific for MBP 85-99 [21,22]. The relative dominance of HLA-DR in these functional studies may relate to the fact that HLA-DR is more strongly expressed by peripheral antigen presenting cells [23]. However, HLA-DQ shows a distinctive pattern of thymic expression, and has been posited to exert an effect through thymic selection [23].

HLA-Tg mice constitute a valuable resource for dissecting the association of disease susceptibility with the specific gene products of the HLA-DR15 haplotype. Studies with HLA class-II Tg mice have demonstrated HLA-DR-dependent disease following immunization by MBP, PLP, or MOG [24-27]. In HLA class-II Tg mice expressing the DRB1*1501 allele of the HLA-DR15 haplotype, the susceptibility to EAE induced by MBP [24] or MOG [27] supports a functional contribution of DRB1*1501 to MS. However, our recent studies in HLA-DR15-Tg mice (DRB1*1501, DQB1*0602) show that susceptibility to MOBP is determined by HLA-DQB1*0602, and not by the DRB1*1501 product of the HLA-DR15 haplotype [28]. These studies were the first to functionally implicate DQ6-associated autoimmunity in the pathogenesis of MS and to offer a mechanism for DQB1*0602 as a disease-predisposing allele, in contrast to some human and transgenic mouse studies suggesting a protective role for HLA-DQ6 [29-31].

While our recent study [28] showing that MOBP can induce MS-like disease in HLA-DQB1*0602-Tg mice offers a rationale for the HLA-DQB1*0602 association with MS, it raises the question of whether DQ6-autoimmunity against other myelin/neuronal target antigens/epitopes can be pathogenic and may also play a role in pathogenesis of MS. PLP autoimmunity has been strongly implicated in some functional studies attempting to correlate immune reactivity with MS relapse [32]. We therefore investigated the pathogenic potential of PLP in HLA-Tg mice expressing the DRB1*1501 and DQB1*0602 gene products of the HLA-DR15 haplotype. We show that, rather strikingly, the disease-susceptibility to the one of the most prominent encephalitogenic target antigen implicated in human MS, is determined by DQB1*0602, and not by DRB1*1501. These findings suggest a mechanism for the involvement of HLA-DQB1*0602-associated pathogenic autoimmunity in the pathogenesis of MS, implicating the HLA-DQB1*0602 in the genetic susceptibility to MS.

## Methods

### Mice

HLA-DR15 (DRA1*0101;DRB1*1501) and control (DRA1*0101; DRB1*1502)-Tg mice (MHC-II^-/-^) (referred to here as DRB1*1501-Tg and DRB1*1502-Tg, respectively) were generated by Dr. Chella David [29]. The HLA-DQ6 (DQA1*0102;DQB1*0602)-Tg mice (MHC-II^-/-^) (referred to here as DQB1*0602-Tg), were generated by Dr. Danny Altmann as described previously [33]. HLA-DQ6 transgenic mice were crossed to an H-2Aβ^-/- ^line [34] and maintained thereafter as homozygous knockouts for H-2Aβ. The expression level of HLA-DRB1*1501 and HLA-DQB1*0602 heterodimers on peripheral B cells from DRB1*1501- and DQB1*0602-Tg mice, respectively, was as previously shown by us [28]. Both DRB1*1501-Tg and DQB1*0602-Tg (and also control DRB1*1502-Tg) mice were maintained as homozygous lines at The Weizmann Institute SPF animal facility. All animal procedures and experiments were approved by the IACUC at The Weizmann Institute

### Recombinant human ΔPLP and PLP synthetic peptides

**Figure 1:**
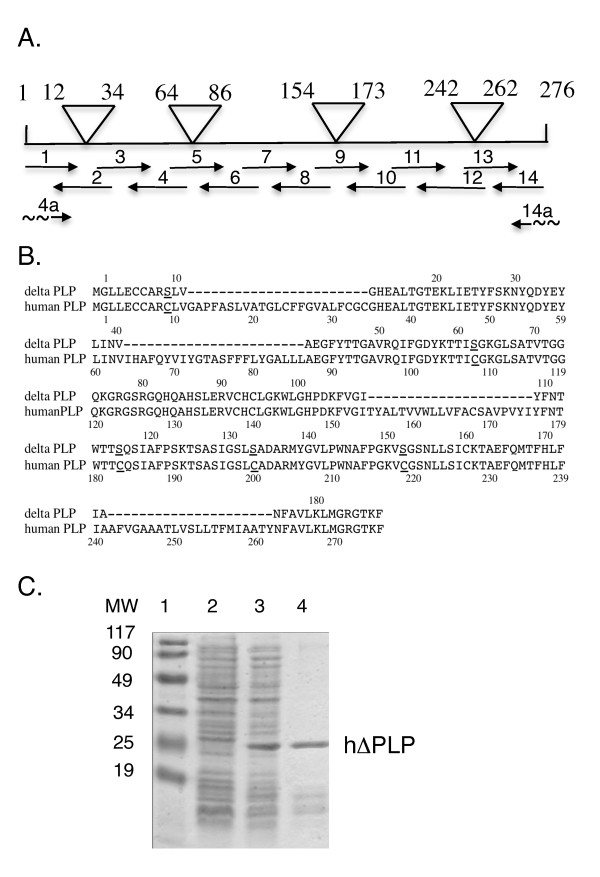
**Preparation of recombinant hΔPLP**. (**A**) Scheme for the preparation and amplification of the synthetic hΔPLP DNA template, which is devoid of the hydrophobic putative transmembrane domains of hPLP, represented by the upper triangles, using the PCR overlap extension technique. (**B**) Alignment of deduced amino acid sequence of hΔPLP with full length native human PLP. The hΔPLP amino acid sequence derived from the DNA sequence of the pRSET/hΔPLP. The dot line in the hΔPLP sequence indicates the positions of the deleted hydrophobic domains. Underlined C and S represent the cysteine to serine substitutions introduced to minimizing incorrect refolding and to increase solubility of the expressed protein. (**C**) Coomassie Brilliant Blue-stained gel of the expressed and purified hΔPLP from bacteria; lane 1, bacterial host proteins before IPTG induction (20 μg of cellular proteins); lane 2, bacterial proteins after IPTG induction (20 μg cellular proteins; lane 3, hΔPLP (~1.5 μg) purified by metal chelate affinity chromatography on Ni^2+^-nitrilotriacetic acid agarose.

ΔPLP was constructed to delete the sequences encompassing the hydrophobic putative transmembrane domains of the human PLP (Figure 1A&1B), thus enabling the expression of soluble protein. The ΔPLP was synthetically constructed using overlapping oligomers (depicted in Figure 1A) spanning the whole DNA sequence encoding the ΔPLP (Figure 1B). 50- to 70-nucleotide-long oligonucleotides, which represent codons of the amino acid residues of the aligned ΔPLP, and which are complementary at their 5' and/or 3' ends to their neighboring oligonucleotides by an overlap of 15-18 nucleotides were synthesized by the Weizmann Institute of Science Synthesis Unit. Relevant oligonucleotides include specific restriction endonuclease sites to enable cloning (or in-frame ligation of internal DNA fragment if necessary). The following oligonucleotides - (1) 5'ATGGAATTCGCTAGCATGGGCTTGTTAGAGTGCTGTGCAAGATCACTGGTAGGACATGAAGCC 3';(2) (rev) 5' GTTTTTCGAGAAATAGGTCTCAATTAACTTTTCTGTGCCAGTGAGGGCTTCATGTCCTACCAG 3'; (3) 5' ACCTATTTCTCGAAAAACTACCAAGACTATGAGTATCTCATCAATGTGGCTGAGGGCTTCTAC 3'; (4) (rev) 5' GCCAAAGATCTGACGGACTGCGCCGGTGGTGTAGAAGCCCTCAGCCAC 3'; (5) 5' CAGTCCGTCAGATCTTTGGCGACTACAAGACCACCATCAGCGGCAAGGGCCTGAGCGCAACGGTA 3'; (6) (rev) 5' AGCTTGATGTTGGCCTCTGGAACCCCTCCCCTTCTGGCCCCCTGTTACCGTTGCGCTCAGGCC 3'; (7) 5' AGAGGCCAACATCAAGCTCATTCTTTGGAGCGTGTGTGTCATTGTTTGGGAAAATGGCTGGGA 3'; (8) (rev) 5' GGTGGTCCAGGTGTTGAAGTAAATGCCCACAAACTTGTCTGGATGTCCCAGCCATTTTCCCAA 3'; (9) 5' TTCAACACCTGGACCACCAGCCAGTCTATTGCCTTCCCAAGCAAGACCTCGGCCAGTATAGGC 3'; (10) (rev) 5' AGCATTCCATGGGAGAACACCATACATTCTGGCGTCAGCAGAGAGACTGCCTATACTGGCCGAGGT 3'; (11) 5' GGTGTTCTCCCATGGAATGCTTTCCCGGGCAAGGTTTCTGGCTCCAACCTTCTGTCCATCAGC 3'; (12) (rev) 5' GTTAGCAATAAACAGGTGGAAGGTCATTTGGAACTCAGCTGTTTTGCTGATGGACAGAAGGTT 3'; (13) 5' CACCTGTTTATTGCTAACTTTGCCGTCCTTAAACTCATGGGCCGTGGCACCAAGTTCCTC 3'; (14) (rev) 5' TCAGGATCCTCACTCGAGGAACTTGGTGCC, 3'; - were used in PCR extension for the synthesis of the ΔPLP template (Figure 1A). The template was amplified by PCR using the (1a) 5' ATGGAATTCGCTAGCATGGGCTTGTTA 3'; (14) 5'TCAGGATCCTCACTCGAGGAACTTGGTGCC3' primers, in which NheI and BamHI restriction sites, respectively, are included. The template synthesis, PCR amplification and cloning of the DNA coding the ΔPLP were carried out as we previously described [35]. The PCR product was cloned into pGEM-T vector (Promega, Madison, WI, USA) was than subcloned into pRSET bacterial expression vector (Invitrogen, San DIEGO, CA, USA) 3' to its 6xHis tag, and sequenced using pRSET specific primers and ΔPLP-specific internal primers to confirm an open reading frame for a fusion protein with the amino acid sequence shown in Figure 1B, preceded by (Met)-Arg-Gly-Ser-(His)_6_-Gly-Met-Ala-Ser. Expression in E. coli and purification of ΔPLP on Ni2+-NTA agarose was carried out as detailed previously by Kerlero de Rosbo et al. [36].

The synthetic overlapping peptides spanning ΔPLP and their amino acid sequence are listed in Table 1. All peptides (at least 80% purity) were synthesized in the laboratory of Prof. M. Fridkin, Department of Organic Chemistry, The Weizmann Institute of Science, using the Fmoc technique with an automated peptide synthesizer (AMS422; Abimed, Langenfeld, Germany).

**Table 1:** List of the synthetic peptides spanning hPLP used in this study

Overlapping human pPLP*	Sequence
1-20	GLLECCARCLVGAPFASLVA
30-51	LFCGCGHEALTGTEKLIETYFS
41-60	GTEKLIETYFSKNYQDYEYL
84-102	LLLAEGFYTTGAVRQIFGD
95-116	AVRQIFGDYKTTICGKGLSATV
107-124	ICGKGLSATVTGGQKGRG
117-139	TGGQKGRGSRGQHQAHSLERVCH
139-151	HCLGKWLGHPDKF
175-194	IYFNTWTTCQSIAFPSKTSA
175-183S-194	IYFNTWTTSQSIAFPSKTSA
185-206	SIAFPSKTSASIGSLCADARMY
195-216	SIGSLCADARMYGVLPWNAFPG
206-226	YGVLPWNAFPGKVCGSNLLSI
215-235	PGKVCGSNLLSICKTAEFQMT
226-245	ICKTAEFQMTFHLFIAAFVG
257-276	MIAATYNFAVLKLMGRGTKF

*, Peptide sequences correspond to native hPLP, except 175-183S-194

### Induction of EAE

Mice were injected subcutaneously at one site in the flank with 200 μl of emulsion containing 200 μg ΔPLP or 200 μg peptide in CFA with 300 μg Mycobacterium tuberculosis H37Ra (Mt.) (Cat. No: 3114-25, Difco Laboratories, Detroit, MI). Mice received 300 ng pertussis toxin (Cat. no. P-9452, Sigma, Saint-Louis, MI) in 500 μl PBS in the tail vein immediately and 48 h after the immunization (Protocol 1). In some cases, as indicated, mice received an identical booster immunization on the flank, one week later (Protocol 2). Following the encephalitogenic challenge, mice were observed daily and clinical manifestations of EAE were scored on a scale of 0-6: 0, no clinical signs; 1, loss of tail tonicity; 2, flaccid tail; 3, hind leg paralysis; 4, hind leg paralysis with hind body paresis; 5, hind and fore leg paralysis; 6, moribund, as previously described [37].

### T cell responses

Mice were immunized with 150 μg ΔPLP or individual peptides emulsified in complete Freund's adjuvant (CFA) containing 150 μg Mycobacterium tuberculosis (Mt) H37Ra (Cat. No:3114-25, Difco Laboratories, Detroit, MI). Ten or 14 days post immunization, draining lymph nodes or spleens, respectively, were removed and cultured in triplicate in the presence or absence of relevant antigens, as previously described [38]. The cultures were incubated for 48-72 h at 37 C in humified air containing 7.5% CO_2_. [^3^H] Thymidine (1 mCi/well) was added for an additional 16 h of incubation and the cultures were then harvested and counted using a Matrix 96 Direct Beta Counter (Packard Instrument, Meriden, CT). The results were expressed as stimulation index (SI; mean cpm of antigen containing cultures/mean c.p.m of medium-containing cultures). In some experiments, as indicated, mice were immunized as for induction of EAE (Protocol 2).

### Cytokine analysis

IL-2, IFN-γ, IL-4, IL-10 and TNF-α were measured by ELISA according to standard protocols from PharMingen (San Diego, CA), as described previously [39]. The capture antibodies were rat anti-mouse IL-4 (18191D; PharMingen), rat anti-mouse IL-2 (18161D; PharMingen), rat anti-mouse IL-10 (AMC0102; BioSource International, Camarillo, CA,) rat anti-mouse IFN-g (AMC4834; BioSource International), and rat anti TNF-α (555052;. PharMingen) The biotinylated antibodies used were rat anti-mouse IL-4 (18042D), rat anti-mouse IL-2 (18172D), rat anti-mouse IL-10 (18152D) and rat anti-mouse IFN-γ (18112D; all from PharMingen). Biotin anti mouse TNF-α (B121372; Biolegend). IL-17 was measured by ELISA using a DuoSet ELISA Development kit (DY421; R&D Systems, Inc., Minneapolis, MN). TGF-β was measured by ELISA according to the standard protocol from R&D Systems (Minneapolis, MN), using recombinant human TGF-β sRII/Fc chimera as capture reagent (341-BR; R&D Systems) and biotinylated anti-human TGF-β1 antibody (BAF240; R&D Systems). Recombinant human TGF-β1 (240-B; R&D Systems) was used to construct the standard curve.

### Pathological examination

Mice were perfused with 4% paraformaldehyde in PBS, and the tissues were post-fixed for 24 h at 4°C. Histological evaluation was performed on paraffin-embedded sections of spinal cords that were sampled 19 days post-immunization as the experiment was terminated. Paraffin sections were stained with H&E and Luxol fast blue to assess inflammation and demyelination respectively. In consecutive sections, immunohistochemistry was performed with Abs directed against the following targets: macrophages/activated microglia (MAC3: BD Pharmingen; Iba-1: Wako-chem, Japan), and T cells (CD3: Chemicon International) [36]. For staining, paraffin sections were pretreated with a steamer for 60 min. Bound primary Ab was detected with a biotin-avidin technique as previously described in detail [36].

## Results

### Immunogenic T cell epitopes of hΔPLP in transgenic mice expressing MS-associated DRB1*1501 and DQB1*0602 products of the HLA-DR15 haplotype

**Figure 2:**
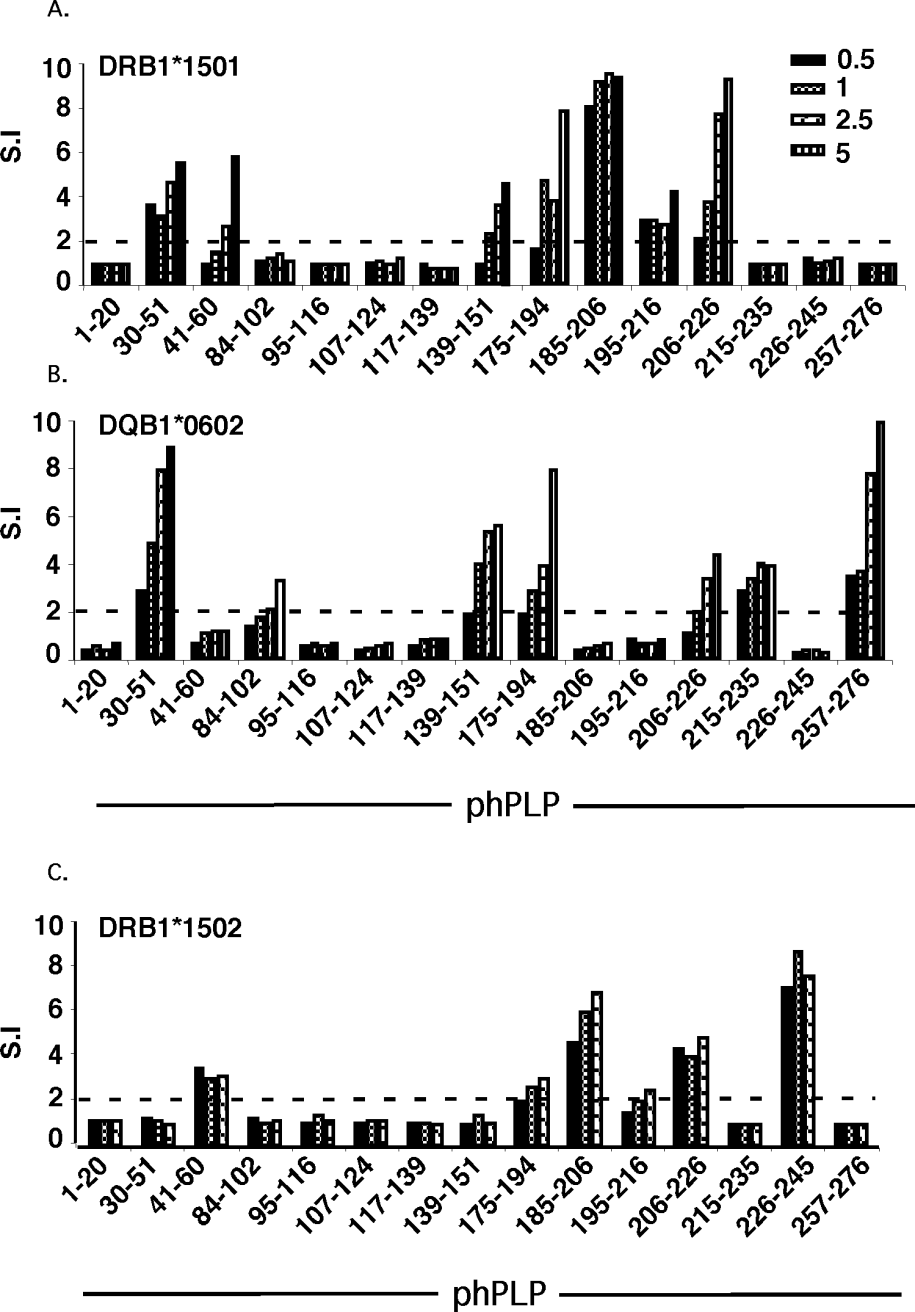
**Mapping immunogenic DR15- and DQ6-associated PLP T-cell epitopes by immunization with individual overlapping peptides spanning hΔPLP**. HLA-DRB1*1501-Tg, HLA-DQB1*0602-Tg and HLA-DRB1*1502-Tg (as control HLA-Tg line) mice were immunized (s.c.) each with an individual peptide (derived from native hPLP) of the overlapping peptides spanning hΔPLP. Ten days later, the primed LNC of immunized mice were analyzed ex-vivo for recall proliferative response to the immunizing peptide (0.5 - 5 μg/ml). Results expressed as stimulation index (S.I., mean cpm of antigen containing cultures/mean c.p.m of medium-containing cultures) are from one experiment with pooled draining LNC from two mice immunized with each individual peptide. Results are representative of three independent experiments.

To define HLA-DR15 haplotype-related immunogenic epitopes of human PLP, DRB1*1501- and DQB1*0602-Tg mice were immunized with each of the individual overlapping peptides spanning the aqueous-soluble recombinant hΔPLP [Δ, deleted of hydrophobic (transmembrane) domains] (listed in Table 1). Ten days later, the primed LNC were analyzed *ex-vivo *for a recall proliferative response to variable concentrations of the immunizing peptide. As shown in Figure 2A, the phPLP30-51, 139-151, 175-194, 185-206, and 206-226 peptides that elicit a significant T-cell response in DRB1*1501-Tg mice are likely to contain DRB1*1501-presented immunogenic eitopes, with phPLP175-194, 185-206, and 206-226 eliciting the strongest DRB1*1501-associated T-cell response. In the DQB1*0602-Tg mice, T-cell responses were seen in response to peptides phPLP30-51, 139-151, 175-194, 215-235, and 257-276 (Figure 2B), with responses to phPLP30-51, 139-151, 175-194, and 257-276 being about equally strong, although the responses to phPLP30-51 and phPLP257-276 were larger.

As a control for the DRB1*1501-Tg mice, we mapped the hΔPLP T-cell epitopes also in DRB1*1502-Tg mice, which differ from HLA-DRB1*1501 only in one amino acid residue [glycine for leucine substitution at position 86 [40]]. This DR15 allele is the predominant one in SE Asia and is rarely associated with MS. As shown in Figure 2C, the immunodominant epitope profile in DRB1*1502-Tg mice differed from that in DRB1*1501-Tg mice. Thus peptides PLP185-206, 206-226, and 226-245 were co-dominant in DRB1*1502-Tg mice, while PLP30-51, 139-151,175-194 epitopes that were immunogenic in DRB1*1501-Tg mice could not elicit T cell responses in DRB1*1502-Tg mice, suggesting the significance of 86Leu in the β chain of DRB1*150 [41] for recognition of PLP epitopes, and the specificity of our peptide immunization.

### Analysis of immunogenic/immunodominant T-cell epitopes following immunization of DRB1*1501- and DQB1*0602-Tg mice with hΔPLP

**Figure 3:**
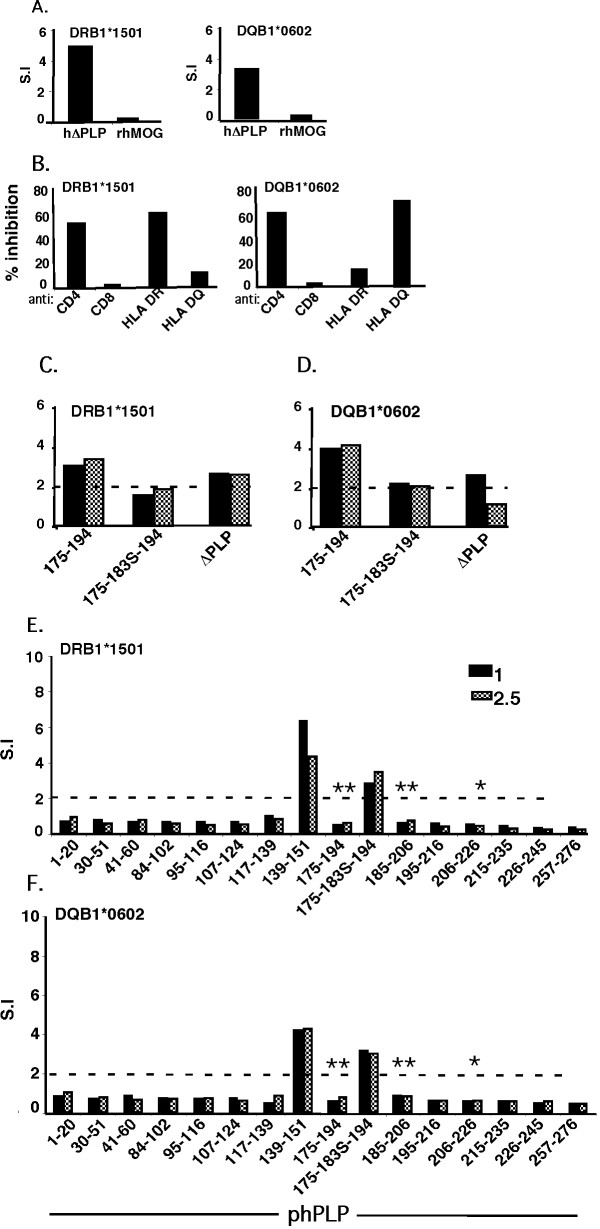
**Epitope-specificity of hΔPLP -primed T-cells**. (**A**) Immunogenicity of hΔPLP in DRB1*1501- and DQB1*0602-Tg mice. HLA-Tg mice (two mice per Tg line) were immunized (s.c.) with 200 μg of hΔPLP in CFA. LNCs from draining LNs obtained 10 later were analyzed ex-vivo for their recall proliferative reponse to 5 μg/ml of hΔPLP or to hMOG (as control recombinant protein). Results (S.I.) are from pooled lymphocytes of each immunized HLA-Tg line. (**B**) *HLA-class II restriction of h*Δ*PLP primed LNCs*. DRB1*1501- and DQB1*0602-hΔPLP-primed lymphocytes from (**A**) were analyzed in-vitro for their recall prolifrative response to hΔPLP in the absence or presence of blocking mAb specific for mouse CD4 (Leaf ™Purified anti-mouse CD4), CD8 (Leaf™ purified anti-mouse CD8a), or specific for human HLA-DR (L243) or HLA-DQ (L2) class-II molecules, or respective isotype control Abs.% inhibition - the S.I. calculated for the recall proliferative response in the presence of neutralizing Abs was divided by the S.I. of the response in the absence of neutralizing Abs (×100). (**C, D**) *Antigenicity of mutated PLP175-194 peptide (PLP175-183S-194)*. DRB1*1501 (C) - and DQB1*0602 (D)- Tg mice (two mice/group) were immunized with 200 μg phPLP175-194 in CFA. Cells from draining LNs obtained 10 days later were analyzed ex-vivo for their recall proliferative response to 1 or 2.5 μg/ml of native phPLP175-194, PLP175-183S-194 mutant peptide, or hΔPLP. The proliferative response was measured as described in Methods. Results (S.I.) are from pooled lymphocytes of immunized HLA- Tg mice. (**E, F**) *Ex-vivo analysis of immunodominant epitopes*. Tg mice (two mice per HLA-Tg line) were immunized with 200 μg of in CFA at the flank, as described in Methods for the induction of EAE (protocol 2). Spleen cells were obtained from each of the immunized mice on day 14 after immunization and cultured in vitro in triplicates in the absence or presence of 1 or 2.5 μg/ml of each of the overlapping peptides (derived from native hPLP) spanning the hΔPLP, and in the presence of PLP175-183S-194 mutant peptide. Results are the mean S.I. of two individual spleens from mice immunized with same peptide in each HLA-Tg line. Results obtained from another independent experiment that was carried out in the same manner showed a similar pattern of reactivity to the overlapping peptides. *, and **, represent native PLP peptides, the cysteines of which were replaced by serine in the hΔPLP, where *, depicts peptides containing Cys within the nonameric core epitope for DRB1*1501 and/or DQB1*0602 molecule, but is not a major TCR or MHC binding residue, as predicted in silico: and **, depicts peptides containing Cys in the nonameric core epitope for DRB1*1501 and/or DQB1*0602 molecule that was predicted in silico to be a major TCR-contact or MHC-contact residue.

To validate the DR15-haplotype-relevant epitopes that had been defined by immunization with the individual peptides and to distinguish which were dominant and which cryptic, we immunized DRB1*1501- and DQB1*0602-Tg mice with aqueous-soluble recombinant hΔPLP (Figure 1). It should be noted that in order to increase the water-solubility of the recombinant hΔPLP, several cysteine residues of the hΔPLP were replaced by serine (Figure 1B, underlined cysteine residues). We indeed obtained a highly water-soluble recombinant hΔPLP, however, some of the cysteines appeared critical for some T cell epitopes, as described below. Figure 3A shows that the hΔPLP preparation was immunogenic for the DRB1*1501- and DQB1*0602-Tg lines, as the mice mounted a significantly higher specific T cell response to hΔPLP than that to control non-relevant recombinant protein, recombinant human MOG (rhMOG), and that the T-cells reactive against the hΔPLP in DRB1*1501- and DQB1*0602-Tg mice were CD4^+ ^and DRB1*1501- or DQB1*0602-restricted, respectively (Figure 3B). Primed LNCs from DRB1*1501- and DQB1*0602-Tg mice that were immunized with hΔPLP/CFA, were analyzed *ex-vivo *for a recall proliferative response to a panel of overlapping peptides spanning the hydrophilic domains of native hPLP, at various concentrations. The results obtained from two independent experiments showed that hΔPLP-primed LNCs of both DRB1*1501- and DQB1*0602-Tg mice reacted to a single epitope only, the PLP139-151 [(Additional file 1: Figure S1). i.e., data from these experiments were similar to the data of Figures 3E&3F without the mutated peptide PLP175-183S-194].

**Figure 4:**
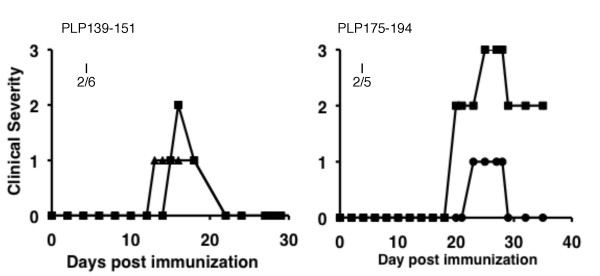
Clinical course of actively induced in HLA-DQB1*0602 Tg mice. EAE was induced by the indicated PLP peptide in CFA as described in methods (protocol 2): Pertussis toxin was administered immediately and 48 h after immunization. The mice received an identical booster immunization with PLP peptide in CFA on the flank one week later. I, incidence.

These results may suggest that PLP139-151 is the immunodominant epitope of PLP for DRB1*1501- or DQB1*0602-Tg mice, and all the other immunogenic epitopes defined by peptide immunization (Figure 2A&2B) are cryptic. However, before drawing a final conclusion that PLP139-151 is indeed the sole immunodominant epitope of PLP for DRB1*1501- or DQB1*0602-Tg mice and all the other immunogenic epitopes defined by peptide immunization (Figure 2A&2B) are cryptic, we have examined whether the replacement of several cysteines by serine in the hΔPLP, had any effects on the immunogenicity of epitopes, as the cysteines in the synthetic overlapping peptides were left intact. Thus, the inability of immunogenic phPLP30-51, 41-60, and 195-216 (Figure 2A), which did not contain cysteine or their corresponding cysteines in hΔPLP were left intact, to stimulate hΔPLP-primed T-cells from DRB1*1501-Tg mice argues that these peptides indeed contain cryptic PLP epitopes for DRB1*1501-Tg mice. Similarly, immunogenic phPLP30-51, 215-235, and 257-276 peptides (Figure 2B), for which their corresponding cysteines in hΔPLP were not replaced to serine, contain cryptic PLP epitopes for DQB1*0602-Tg mice as they were non-stimulatory to hΔPLP-primed cells. The question remains as to whether the immunogenic phPLP175-194, 185-206 and 206-226 peptides (Figures 2B&2C) which have a Cys that was replaced by Ser in hΔPLP and were not stimulatory for hΔPLP-primed T-cells from DRB1*1501- and/or DQB1*0602-Tg mice (indicated by an asterisk(s) in Figures 3E&3F) are also cryptic, or whether the replacement of Cys by Ser affected their immunogenicity. This question is of particular significance for phPLP175-194 that was found to be one of the major encephalitogenic PLP epitopes in DQB1*0602-Tg mice (Table 2, Figure 4).

**Table 2:** Active EAE induction with hPLP peptides

HLA Tg mice	Immunization	Incidence	Maximal clinical severity	Day of onset
DRB1*1501	hPLP30-51	0/4	--	--
	hPLP30-51*	0/4	--	--
	hPLP41-60	0/4	--	--
	hPLP41-60*	0/4	--	--
	hPLP95-116*	0/4	--	--
	hPLP139-151*	0/3	--	--
	hPLP175-194	0/3	--	--
	hPLP175-194*	0/4	--	--
	hPLP185-206	0/3	--	--
	hPLP185-206*	0/9	--	--
	hPLP195-216	0/3	--	--
	hPLP195-216*	0/4	--	--
	hPLP206-226	0/3	--	--
	hPLP206-226*	0/3	--	--
	hPLP257-276*	0/3	--	--
	hΔPLP*	0/4	--	--

DQB1*0602	hPLP30-51	0/2	--	--
	hPLP30-51*	0/4	--	--
	hPLP84-102	0/2	--	--
	hPLP139-151*	6/15	1,2,1,1,1,1	13,15,16,13,14,14
	hPLP175-194*	5/9	2,3,1,3,1	13,14,15,20, 21
	h175-183S-194*	0/5	--	--
	hPLP206-226	0/3	--	--
	hPLP206-226*	0/4	--	--
	hPLP215-235	0/4	--	--
	hPLP215-235*	0/4	--	--
	hPLP257-276	0/8	--	--
	hPLP257-276*	0/4	--	--
	hΔPLP*, **	0/8	--	--

Mice were injected with 200 μg of PLP peptide or hΔPLP, and received 300 ng PT immediately and after 48 h (protocol 1)

*, Mice received a boost after a week (protocol 2)

**, The Cys to Ser replacements in the hΔPLP abrogated the encephalitogeinc potential of epitopes, as shown here for PLP175-183Cys/Ser-194 mutated peptide and detailed in Results

To resolve this question, we first used bioinformatic modeling to predict the nonameric core epitope(s) of the 175-194 region of human PLP with preferred binding mode to DRB1*1501 and/or DQB1*0602 molecules. This modeling [carried out by DR. M. Eisenstein, Chemical Research Support Unit, Weizmann Institute, as previously described for I-A^b^/MOG epitopes [38], but using the known 3D structures of DR15 (PDB -1BX2) and DQ6 (PDB-1UVQ) molecules] predicted the PLP178-NTWTTCQSI-186 as core nonameric epitope of phPLP175-194 for DRB1*1501, and the two overlapping nonameric core epitopes, PLP178-NTWTTCQSI-186 and PLP179-TWTTCQSIA-187, with preferred binding mode to the DQB1*0602 molecule. Furthermore, the cysteine (183C) within each of these nonameric core epitopes was predicted to be at a critical MHC (p6 for PLP178-186) or TCR (p5 for PLP179-187) contact position, suggesting that its replacement by another residue (183S) is likely to affect immunogenicity/antigenicity of the PLP175-194 in DRB1*1501- or DQB1*0602- Tg mice. This possibility was then confirmed experimentally by showing that PLP175-194-primed LNCs from DRB1*1501- or DQB1*0602-Tg mice could be stimulated by the native PLP175-194 peptide, but not by mutated peptide PLP175-183S-194 (Figures 3C&3D). Moreover, *ex-vivo *analysis of the recall proliferative response of hΔPLP-primed LNCs of both DRB1*1501- and DQB1*0602-Tg mice to a panel of the overlapping peptides spanning the hydrophilic domains of native hPLP, as well as to the mutated peptide PLP175-183S-194 comprising the 183C > S replacement in the hΔPLP, showed a significant reactivity against PLP139-151 and also against mutated PLP175-183S-194 peptide, but not against the native PLP175-194 peptide (Figures 3E&3F).

The in-silico analysis, together with the experimental data (Figures 3C-F), suggests that the encephalitogenic PLP175-194 epitope is unlikely to be cryptic, and that PLP139-151 and PLP175-194 are the co-dominant epitopes of PLP for both DRB1*1501- and DQB1*0602-Tg mice. These data also suggest that contrary to consensus, the Cys to Ser replacement may not be inert for immunogenicity/antigenicity of T-cell epitopes.

### DQB1*0602- but not DRB1*1501-Tg mice are susceptible to PLP-induced EAE

The HLA-Tg lines were immunized for induction of EAE with each of the hPLP peptides that harbor T-cell epitope for each relevant HLA-Tg line, as defined in Figure 2, regardless of whether some of them were suggested to contain cryptic epitopes for DRB1*1501-Tg mice (phPLP30-51, 41-60, and 195-216) or for DQB1*0602-Tg mice (phPLP30-51, 215-235, and 257-276) (as detailed above). The results summarized in Table 2 show that inoculation using two encephalitogenic protocols (see Methods) with any of the DRB1*1501-immunogenic peptides did not cause clinical EAE in DRB1*1501-Tg mice. The phPLP95-116 which overlaps with phPLP91-110 that was reported to be encephalitogenic for HLA-DR3 mice [26] was also non-encephalitogenic in DRB1*1501-Tg mice. In contrast, the encephalitogenic inoculation with the DQB1*0602-immunogenic peptides resulted in the development of overt clinical EAE in DQB1*0602-Tg mice by phPLP139-151 and phPLP175-194 (Table 2). The clinical course of EAE induced in HLA-DQB1*0602-Tg mice by phPLP139-151 (Figure 4A) or by phPLP175-194 (Figure 4B) presented with clinical manifestations typical of classical EAE with caudo-rostral ascending paralysis that developed about 2 weeks after immunization. Notably, the Cys to Ser mutation in PLP175-183S-194 peptide abrogated the encephalitogenic potential of phPLP175-194 in the DQB1*0602 Tg mice (Table 2), explaining in part the failure of the immunization with whole hΔPLP (containing Cys to Ser replacements) to induce EAE in DQB1*0602 Tg mice (Table 2).

### Differential cytokine profile of DRB1*1501- and DQB1*0602-derived PLP139-151- and PLP175-194-reactive T cells

**Figure 5:**
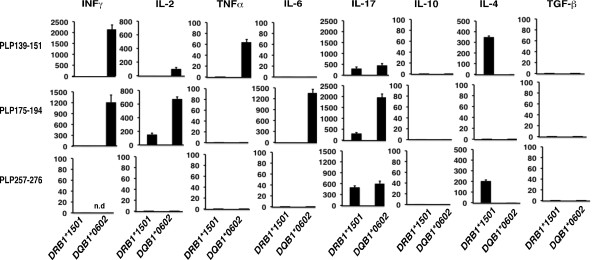
Cytokine secretion profile of phPLP139-151, 175-194, and 257-276-primed LNC derived from immunized DRB1*1501- or DQB1*0602-Tg mice. HLA-DRB1*1501- and DQB1*0602-Tg mice were immunized by s.c. injection of 150 μg phPLP139-151, phPLP175-194 or phPLP257-276 (as control) in CFA. Ten days later, draining LNC (pooled from 3 mice) were cultured for 48 h without or with the immunizing peptide, respectively, and supernatants were analyzed for the secretion of indicated cytokines. Values presented (pg/ml) are after background (without antigen) cytokines were subtracted. The results represent three experiments done in triplicates, and are the mean cytokine concentration ± SE.

The finding that phPLP139-151 and phPLP175-194 were encephalitogenic only in DQB1*0602 but not in DRB1*1501 Tg mice, despite their ability to stimulate quantitatively similar T cell responses in both Tg lines demanded further investigation. We therefore analyzed the Th1/Th2/Th17 cytokine profiles associated with the T-cell reactivity to phPLP139-151 and phPLP175-194 by both Tg lines. The phPLP139-151- and phPLP175-194-primed LNCs derived from DRB1*1501- or DQB1*0602-Tg mice were stimulated ex-vivo with the relevant priming peptide, and cytokines secreted into the supernatants were analyzed. Figure 5 shows that reactivity against phPLP139-151 and phPLP175-194 in DQB1*0602-Tg mice was explicitly pro-inflammatory, with variably high secretion of IL-2, IFNγ, IL-17 and TNFα by PLP139-151- or PLP175-194-reactive T-cells, respectively, and relatively low IL-4 and IL-10. This Th1/Th17 cytokine profile (Figure 5) correlates with the encephalitogenic capacity of phPLP139-151 and phPLP175-194 in-DQB1*0602-Tg mice (Table 2, Figure 4). Moreover, the consistently lower secretion of IL-17 and IL-2 by phPLP139-151- compared to phPLP175-194- primed LNCs derived from DQB1*0602-Tg mice, is consistent with the lower encephalitogenic potential of phPLP139-151 compared to phPLP175-194 in DQB1*0602-Tg mice (Table 2 &Figure 4). In contrast to DQB1*0602-Tg mice, the cytokine profile of the DRB1*1501-derived primed LNCs against the relevant priming peptide, phPLP139-151 or phPLP175-194, was of a more anti-inflammatory type, with low IL-2, IFNγ, TNFα and IL-17, and with secretion of IL-4 by PLP139-151-reactive T-cells (Figure 5). Thus, the Th2 phenotype of the phPLP139-151-reactive T-cells, and the very low secretion of pro-inflammatory cytokines by phPLP175-194-reactive T-cells derived from DRB1*1501-Tg mice may explain the relative resistance of these mice to EAE induction by phPLP139-151 or by phPLP175-194.

Taken together, these patterns of cytokine secretion suggest that PLP139-151- and PLP175-194-reactive T-cells are directed to a Th1/Th17 phenotype in EAE-susceptible DQB1*0602-Tg mice and are more Th2 in nature in EAE-resistant DRB1*1501-Tg mice.

### Pathology of PLP-induced EAE in HLA-DQ6-Tg mice

**Figure 6:**
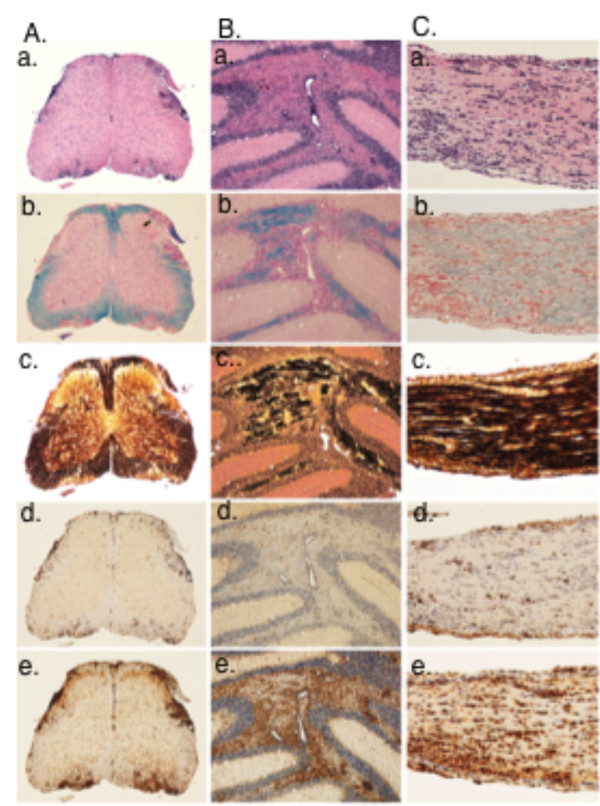
Histopathology of PLP-induced EAE in HLA DQB1*0602 Tg mouse. Samples were taken on day19 after immunization. Panel (**A**) shows the spinal cord, (**B**) the cerebellum and (**C**) the optic nerve. The sections were stained with hematoxylin & eosin (**a**), Luxol fast blue for myelin. (**b**) Bieschowsky silver impregnation for axons (**c**) and by immunocytochemistry for CD3 (**d**), and Mac-3 (**e**). Note the unusually profound inflammation in the cortex and optic nerve. Original magnifications: A and B: × 25; C: × 100.

We analyzed the histopathology associated with the development of EAE induced in DQB1*0602-Tg mice by phPLP175-194, as the more potent encephalitogenic peptide compared to phPLP139-151. The histopathological analysis of the spinal cord, brain and optic nerves show, gross pathological changes typical of classical EAE, consistent of inflammation (Figure 6a, d, e), demyelination (Figure 6b) and axonal loss (Figure 6c). Inflammatory infiltrates were composed mainly of CD3 positive T-cells (Figure 6Ad, Bd, Cd) and macrophages (Figure 6Ae, Be, Ce). In addition, profound microglia activation was seen in affected brain and spinal cord regions (Figure 6Ae, Be) and optic nerve (Figure 6Ce). An unusual finding was the profound involvement of the brain (shown here in the cerebellum and optic nerves) in comparison to the spinal cord. Generally, EAE induced by PLP or MOG in wild-type mice, the disease mainly affects the spinal cord, and with increasing disease severity there is an additional involvement of CNS region, but a gradient remains with the most severe lesions in the spinal cord compared to other regions of the CNS. Pathological analysis of phPLP175-194-induced EAE in DQ6-Tg mice (Figure 6) shows severe brain involvement [cerebellum (Figure 6B) and optic nerves (Figure 6C)] exceeding that of spinal cord involvement (Figure 6A). Inflammatory infiltrates were composed mainly of CD3 positive T-cells (Figure 6Ad, Bd, Cd) and macrophages Figure 6Ae, Be, Ce). In addition, profound microglia activation was seen in affected brain and spinal cord regions (Figure 6Ae, Be) and optic nerve (Figure 6Ce). Inflammation was also associated with a variably extent of demyelination and acute axonal injury (Figure 6a-c in A-C). Goverman and colleagues [42] have suggested that strong involvement of the cerebellum and brainstem is a feature of Th17-driven disease, which is in line with the high Th17 secretion by PLP175-194-primed LNC derived from DQB1*0602-Tg mice (Figure 5).

## Discussion

In complex trait genetics in general and, notably the autoimmune diseases, there is a fast approaching challenge of how to move from gene-wide association and fine-mapping studies to functional pathways and therapeutics. MS is a case in point: genetic studies identify HLA-DRB1*1501 as a primary risk factor in MS, although the close genetic proximity of HLA-DQB1*0602 and strong linkage disequilibrium between the two make it almost impossible to distinguish the contribution of these genes except through functional studies. Most functional studies relating to HLA products in MS have focused on DRB1*1501, with little consideration for the potential contribution of DQB1*0602-associated autoimmunity to pathogenesis. Our recent studies in HLA-DR15-Tg mice showing that susceptibility to MOBP-induced EAE is determined by HLA-DQB1*0602, and not by DRB1*1501 [28], were the first to implicate DQB1*0602-associated autoimmunity in the pathogenesis of MS and to suggest DQ6 as an important disease predisposing, rather than just a disease-modifying allele, as previously suggested [30]. We now show that the DQB1*0602-associated pathogenic autoimmunity against MOBP is not a limited case, and that DQB1*0602-autoimmunity against other CNS antigens may also play a role in pathogenesis of MS. We show that disease-susceptibility to PLP, a highly encephalitogenic protein and one of the most prominent and 'MS-implicated' antigens in the CNS, is also determined by DQB1*0602, and not by DRB1*1501 gene products of the HLA-DR15 haplotype. Thus, HLA-DRB1*1501 transgenics were found to be refractory to PLP disease induction, whereas the HLA-DQB1*0602 transgenics were susceptible, via T-cells reactive against PLP139-151 and PLP175-194 encephalitogenic epitopes. These findings have important bearing on the candidacy of the DQB1*0602 allele as genetic risk factor for MS.

Analysis of the T-cell autoimmunity against PLP, which showed that PLP is immunogenic for HLA-DRB1*1501- and HLA-DQB1*0602-Tg mice also revealed that the T-cell autoimmunity against PLP in both transgenics is predominantly directed against PLP139-151 and PLP175-194 epitopes, which are the immunodominant and encephalitogenic epitopes for SJL/J mice [43]. However, although both transgenics could mount CD4+ T-cells reactive against these co-dominant PLP epitopes, the PLP139-151 and PLP175-194-reactive T-cells that developed in DQB1*0602-Tg mice were of pathogenic Th1/Th17-type cells that caused the development of clinical EAE in DQB1*0602-Tg mice, while the PLP139-151 and PLP175-194-reactive T-cells were of a more Th2-type that conferred resistance to PLP-EAE in DRB1*1501-Tg mice. Further investigation is required to understand how recognition of the same epitopes in the context of DQB1*0602 class-II molecule drives Th1/Th17 pathogenic T-cell autoimmunity, while the recognition of same epitopes in the context of DRB1*1501 class-II molecule drives more Th2 type autoimmunity. These findings in the reductionist transgenic models are quite challenging since both DRB1*1501 and DQB1*0602 molecules are co-expressed in HLA-DR15+ MS patients, raising the question on whether the net T-cell autoimmunity against PLP, and particularly against the PLP139-151 and PLP175-194 epitopes, in HLA-DR15+ MS would be of Th1/Th17 pathogenic autoimmunity or more of anti-inflammatory Th2 type. This question can be resolved by analyzing the Th1/Th17/Th2-type of T-cell reactivity against PLP, particularly against PLP139-151 and PLP175-194 epitopes by PBLs of HLA-DR15+ MS patients, compared to the Th1/Th17/Th2-type response by their DRB1*1501- and DQB1*0602-restricted T-cells reactive against these epitopes. In this context, however, it is worth mentioning that (DRB1*1501 × DQB1*0602)F1 double Tg mice, expressing both DRB1*1501 and DQB1*0602 molecules were recently found to be resistant to EAE induction by either PLP139-151 or PLP175-194 epitope (Kaushansky et al., unpublished data). Why and by what mechanisms the susceptibility to EAE induced by these immunodominant epitopes was not inherited in the F1 double-Tg mice as a dominant trait, as is usually the case for EAE susceptibility to other encephalitogenic antigens/epitopes in laboratory animals, is now under investigation in our laboratory.

The central question of whether HLA-DRB1*1501, HLA-DQB1*0602, or their co-expression is the primary risk factor in MS has not been fully resolved by genetic studies due to their linkage disequilibrium. Although most MS genetics studies in Caucasian populations suggest HLA-DRB1*1501 as the genetic risk factor in MS [13-15], several other genetic studies in unique populations suggest that HLA-DQ alleles may also be a risk factor. In a small cohort of Norwegian MS patients, some patients were identified who carried DQB1*0602 or DQB1*0603 without DRB1*1501, but none who were DRB1*1501 without DQB1*0602 [17]. Related observations were made in a relatively small sample of Hong Kong Chinese patients with MS. In that population, DR15 is expressed without DQB1*0602 and DQB1*0602 without DR15, so that one can ask whether either or both of the dissociated genes appear to confer an enhanced risk. It was found that the enhanced risk was associated with DQB1*0602 [16]. A similar case has been made for susceptibility in Afro-Brazilians, where the frequency of DQB1*0602 among patients is higher than that for the main DR15 allele, DRB1*1503, in that unique population [44]. A caveat here is the analysis of HLA-DR and -DQ associations conducted in a large cohort of African American MS patients, which showed a selective association with HLA-DRB1*1501 and not with HLA-DQB1*0602 was identified [18]. Thus, while primacy of HLA-DRB1*15 or HLA-DQB1*0602 has not been conclusive in the African populations that show greater haplotypic diversity than Europeans and distinct patterns of linkage disequilibrium, a potential contribution of HLA-DQB1*0602 to MS susceptibility could still be inferred. The possibility that both HLA-DRB1*1501 and HLA-DQB1*0602 loci influence susceptibility to MS through epistatic interactions has been demonstrated in Canadian MS cohorts, where the HLA-DQA1*0102, which showed no independent association, was found to interact strongly with HLA-DRB1*1501 in trans, increasing MS risk in the presence of HLA-DRB1*1501 and playing a protective role in its absence [45]. Nonetheless, our data showing pathogenic DQB1*0602-associated autoimmunity against MOBP in HLA-Tg mice [28] and against PLP (this study) offer a rationale and potential mechanisms for the HLA-DQB1*0602 association with MS.

The reductionist experiment of transgenic lines, separating HLA-DR and HLA-DQ, offers a chance to dissociate these presentation experiments in a genetic setup rarely seen in humans because of the rarity of recombination events between these loci. Studies with HLA class-II Tg mice have previously demonstrated HLA-DR-dependent disease induced by MBP, PLP, and MOG [24-27]. However, while susceptibility to MBP- and MOG-induced EAE in HLA-Tg mice was determined by the DRB1*1501 allele of the HLA-DR15 haplotype, the previously reported susceptibility to PLP91-110 epitope was associated with the DRB1*0301, a non-Caucasian MS-associated allelic gene of the HLA-DR3 haplotype. Our results show that, unlike the previously reported HLA-DR-dependent susceptibility to MBP-, PLP-, or MOG-induced EAE, the pathogenic autoimmunity against PLP, as well as against MOBP [28], was dependent on HLA-DQB1*0602 rather than HLA-DRB1*1501. This DQB1*0602-associated susceptibility is in striking contrast with human and transgenic mouse studies suggesting a protective role for HLA-DQ6. The closely related allele DQB1*0601, most typically found in South Asian populations, has been reported in two human studies to be associated with protection from MS [31,46]. In accordance with those findings, studies in HLA-Tg-mice [29,30] argued that the presence of HLA-DQB1*0601 could exert an epistatic protective effect on HLA-DR-dependent, anti-myelin autoimmunity. Thus, while HLA-DRB1*1502 Tg mice were susceptible to MOG-induced EAE, the HLA(DRB1*1502 × DQB1*0601) double-Tg mice were resistant [29]. The protective effect of HLA-DQ6(DQA1*0103/DQB1*0601) was reported in more detail in PLP-induced EAE, in which HLA-DR3(DRB1*0301)-Tg mice were susceptible to PLP91-110-induced disease, whereas the HLA-DR3(DRB1*0301) × DQ6(DQB1*0601) double-Tg mice were protected [30]. In our studies, using the Caucasian MS-associated allelic genes (of the HLA-DR15 haplotype) we show the opposite scenario for the role of the HLA-DQ6 in anti-myelin autoimmunity in HLA-Tg mice, as the HLA-DQB1*0602 gene product determined susceptibility to PLP- as well as to MOBP-induced EAE.

The PLP-induced EAE in HLA-DQB1*0602 transgenic mice showed a typical caudo-rostral clinical progression that was associated with CNS demyelination, axonal damage and with optic neuritis. However, unlike usually observed in PLP-induced disease or other EAE models in the wild type mice, the CNS pathology in phPLP175-194-induced EAE in HLA-DQB1*0602 transgenics was more pronounced in the brain rather than in the spinal cord. Such a strong involvement of cerebellum and brainstem exceeding that of spinal cord, which has been suggested by Stromnes et al. [42] to be a feature of Th17-driven disease, corresponded to the high Th17 secretion by PLP175-194-primed LNC derived from DQB1*0602-Tg mice. It should be noted that the DRB1*1501-Tg line (originated from C. David and maintained in our animal facility as homozygotic line for several years) which is refractory to PLP139-151- and to PLP175-194-induced EAE, or to MOBP15-36- induced EAE [28], is susceptible in our hands to EAE induced by MOG35-55 and to MBP89-104 (data not shown), indicating that the disease-resistance or -susceptibility is the result of a selective DRB1*1501-associated Th2 autoimmunity, and DQB1*0602-associated Th1/Th17 autoimmunity, against PLP and MOBP in the setting of the these HLA-humanized mice. Whether such a selective preference for Th2 autoimmunity against PLP or MOBP in the context of DRB1*1501, and Th1/Th17 autoimmunity in the context of DQB1*0602, occurs also in HLA-DR15+ MS should be examined by ex-vivo analysis the patients' responses to these myelin antigen/epitopes in the context of DRB1*1501- and DQB1*0602-associated antigen presentation.

Over the last three decades, autoimmunity against MBP, PLP, and more recently also against other CNS myelin proteins, such as MOG, MOBP and OSP, has been extensively investigated in MS patients, HLA class-II Tg mice, and in wild-type mice, as major target antigens in MS. Clearly we do not yet know the full extent of CNS proteins that may become autoimmune targets in MS, but the credentials of PLP as a target, both in human MS and mouse models are very compelling. When comparing PLP and MBP, PLP is stronger and dominant encephalitogen at least in some EAE models, particularly in SJL/J mice where PLP139-151 and PLP175-194 are the co-dominant encephalitogenic PLP epitopes [43]. TCR^PLP ^transgenic mice on the SJL/J background develop spontaneous EAE with a relatively high frequency [47] compared with TCR^MBP ^transgenic mice on B10.PL background [48]. Further, upon EAE induction with whole spinal cord homogenate, the dominant T cell response is directed against PLP139-151 [49]. These PLP encephalitogenic peptides are also seen in MS patients' responses. A study on high avidity myelin specific T cells in MS documented that both PLP139-151 and the PLP178-191 epitopes are key targets of high avidity T-cell allels and clearly elevated in MS versus healthy controls [50]. Perhaps most tellingly, a clinical/MRI relapse triggered in a patient as consequence of an altered peptide ligand (APL) clinical trial was temporally correlated with spread of their response to PLP 190-209 [51]. Hence, the potential contribution of the autoimmunity against PLP to the pathogenesis of MS, which is likely to be DQB1*0602-associated in HLA-DR15+ MS, should be quite significant.

## Conclusions

Overall, our results showing that DQB1*0602 but not DRB1*1501 determines pathogenic autoimmunity against PLP as well as against MOBP [28] in the HLA-Tg mice suggest a differential, functional role for DQB1*0602 as a predisposing allele in MS, to a greater extent than previously perceived. This places MS more firmly in the group of autoimmune diseases in which a functional association is presumed, including type I diabetes and celiac disease [52,53]. The findings showing that DQB1*0602 determines pathogenic autoimmunity against PLP and MOBP [28], together with previously reported studies showing that DRB1*1501 determines pathogenic autoimmunity against MBP and MOG [24,27] suggest a more complex and differential genetic predisposition to HLA-DR15+ MS, depending on the primary CNS target antigen/epitope against which the pathogenic autoimmunity is primarily directed, or triggered (genotype/antigen/phenotype relationship). Thus, in view of the complex anti-CNS autoimmunity associated with MS and the divergent patterns of clinical manifestation of MS, further work using complementary studies of reductionist transgenic models with the more complex analysis of patient responses will be necessary to elucidate these patterns and potential 'genotype/target Ag(epitope)/phenotype' relationship. This is essential for devising immune-specific therapy as well as for gaining new insights into the etiology of MS. It is also of immense significance for many other areas of endeavor in MS research, from analysis and immune-specific targeting of pathogenic TCR [54] to peptide immunotherapies [55] and programs tracking patient responses [56] for more effective treatment protocols.

## Abbreviations

MS: Multiple sclerosis; EAE: Experimental autoimmune encephalomyelitis; MBP: Myelin basic protein; PLP: Proteolipid protein; MOG: Myelin oligodendrocyte glycoprotein: OSP: Oligodendrocyte-specific protein; MOBP: Myelin-associated oligodendrocytic basic protein; hΔPLP: Recombinant soluble PLP: Deleted of putative hydrophobic domains; *Mt: Mycrobacterium tuberculosis*; SI: Stimulation index; Tg: Transgenic; PT: Pertussis toxin; CFA: Complete Freund's adjuvant.

## Competing interests

The authors declare that they have no competing interests.

## Authors' contributions

NK, ABN, conceived and designed experiments; NK, performed research experiments; NK, ABN, DMA, prepared manuscript; DMA, CSD provided HLA Tg mice; HL, analyzed the pathology. All authors read and approved the final manuscript.

## Supplementary Material

Additional file 1 **Figure S1**. Epitope-specificity of hΔPLP -primed T-cells derived from DRB1*1501- and DQB1*0602-Tg mice: Ex-vivo recall proliferative response to overlapping human PLP peptides. HLA-DRB1*1501- and DQB1*0602- Tg mice (two mice per Tg line) were immunized (s.c.) in the flank with 200 μg of hΔPLP in CFA for induction of EAE (as described in Methods, protocol 2). Spleen cells were obtained on day 14 after immunization and cultured in vitro in triplicates in the absence or presence of 1 or 2.5 μg/ml of each of the overlapping peptides for the ex-vivo analysis of the recall proliferative response to a panel of overlapping peptides spanning the hydrophilic domains of native hPLP. The hydrophilic domains of native hPLP correspond to the hΔPLP, except that in the hΔPLP some of the Cys of the native PLP were replaced by Ser (see Figure 1) to increase solubility. Results are the mean S.I. of two individual spleens from mice immunized with same peptide in each HLA-Tg line. *, and **, represent native PLP peptides, the cysteines of which were replaced by serine in the hΔPLP, where *, depicts peptides containing Cys within the nonameric core epitope for DRB1*1501 and/or DQB1*0602 molecule, but is not a major TCR or MHC binding residue, as predicted in silico: and **, depicts peptides containing Cys in the nonameric core epitope for DRB1*1501 and/or DQB1*0602 molecule that was predicted in silico to be a major TCR-contact or MHC-contact residue. Click here for file
