# Supplementary material for: DQB1*0602 rather than DRB1*1501 confers susceptibility to multiple sclerosis-like disease induced by proteolipid protein (PLP)
Source: J Neuroinflammation. 2012 Feb 8;9:29. doi: 10.1186/1742-2094-9-29 (PMC3344688; doi:10.1186/1742-2094-9-29)
Supplement: Additional file 1 — Figure S1. Epitope-specificity of hΔPLP -primed T-cells derived from DRB1*1501- and DQB1*0602-Tg mice: Ex-vivo recall proliferative response to overlapping human PLP peptides. HLA-DRB1*1501- and DQB1*0602- Tg mice (two mice per Tg line) were immunized (s.c.) in the flank with 200 μg of hΔPLP in CFA for induction of EAE (as described in Methods, protocol 2). Spleen cells were obtained on day 14 after immunization and cultured in vitro in triplicates in the absence or presence of 1 or 2.5 μg/ml of each of the overlapping peptides for the ex-vivo analysis of the recall proliferative response to a panel of overlapping peptides spanning the hydrophilic domains of native hPLP. The hydrophilic domains of native hPLP correspond to the hΔPLP, except that in the hΔPLP some of the Cys of the native PLP were replaced by Ser (see Figure 1) to increase solubility. Results are the mean S.I. of two individual spleens from mice immunized with same peptide in each HLA-Tg line. *, and **, represent native PLP peptides, the cysteines of which were replaced by serine in the hΔPLP, where *, depicts peptides containing Cys within the nonameric core epitope for DRB1*1501 and/or DQB1*0602 molecule, but is not a major TCR or MHC binding residue, as predicted in silico: and **, depicts peptides containing Cys in the nonameric core epitope for DRB1*1501 and/or DQB1*0602 molecule that was predicted in silico to be a major TCR-contact or MHC-contact residue. [file 1742-2094-9-29-S1.PDF]

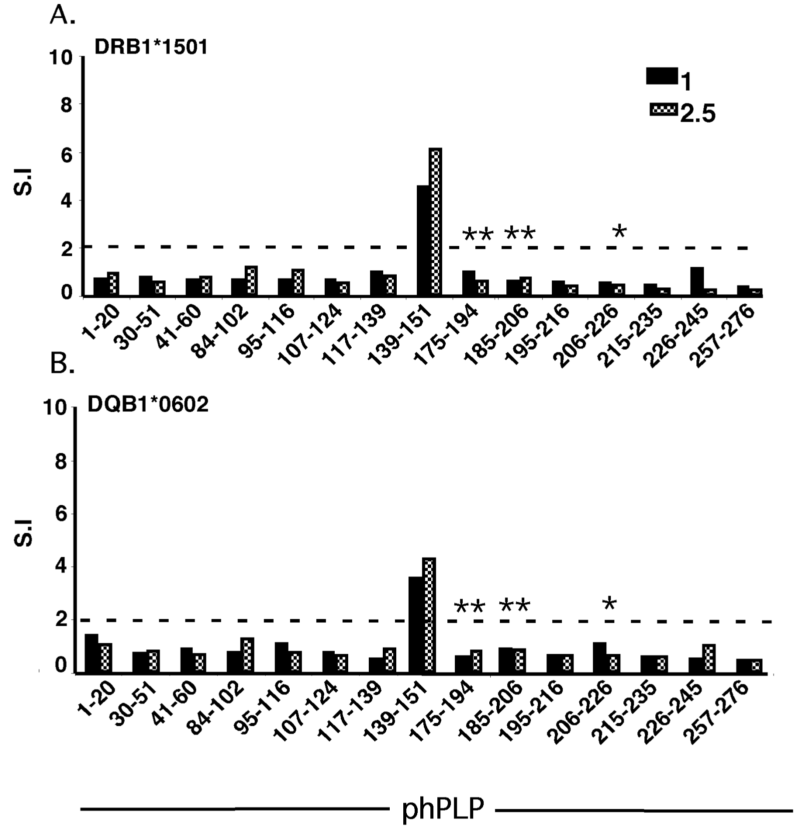

FIGURE S1: Epitope-specificity of hΔPLP -primed T-cells derived from DRB1*1501- and DQB1*0602-Tg mice: Ex-vivo recall proliferative response to overlapping human PLP peptides. HLA-DRB1*1501- and DQB1*0602- Tg mice (two mice per Tg line) were immunized (s.c.) in the flank with 200 μg of hΔPLP in CFA for induction of EAE (as described in Methods, protocol 2). Spleen cells were obtained on day 14 after immunization and cultured in vitro in triplicates in the absence or presence of 1 or 2.5 μg/ml of each of the overlapping peptides for the ex-vivo analysis of the recall proliferative response to a panel of overlapping peptides spanning the hydrophilic domains of <u>native hPLP</u>. The hydrophilic domains of <u>native hPLP</u> correspond to the hΔPLP, except that in the hΔPLP some of the Cys of the native PLP were replaced by Ser (see Fig.1) to increase solubility. Results are the mean S.I. of two individual spleens from mice immunized with same peptide in each HLA-Tg line.

*, and **, represent native PLP peptides, the cysteines of which were replaced by serine in the hΔPLP, where *, depicts peptides containing Cys within the nonameric core epitope for DRB1*1501 and/or DQB1*0602 molecule, but is not a major TCR or MHC binding residue, as predicted in silico: and **, depicts peptides containing Cys in the nonameric core epitope for DRB1*1501 and/or DQB1*0602 molecule that was predicted in silico to be a major TCR-contact or MHC-contact residue.
